# Prospective Prediction of Juvenile Homicide/Attempted Homicide among Early-Onset Juvenile Offenders

**DOI:** 10.3390/ijerph14020197

**Published:** 2017-02-16

**Authors:** Michael T. Baglivio, Kevin T. Wolff

**Affiliations:** 1 G4S Youth Services, LLC, Tampa, FL 33634, USA; 2 Department of Criminal Justice, John Jay College of Criminal Justice, City University of New York, New York, NY 10019, USA; kwolff@jjay.cuny.edu

**Keywords:** homicide, murder, juvenile offending, violence

## Abstract

While homicide perpetrated by juveniles is a relatively rare occurrence, between 2010 and 2014, approximately 7%–8% of all murders involved a juvenile offender. Unfortunately, few studies have prospectively examined the predictors of homicide offending, with none examining first-time murder among a sample of adjudicated male and female youth. The current study employed data on 5908 juvenile offenders (70% male, 45% Black) first arrested at the age of 12 or younger to prospectively examine predictors of an arrest for homicide/attempted homicide by the age of 18. Among these early-onset offenders, males, Black youth, those living in households with family members with a history of mental illness, those engaging in self-mutilation, and those with elevated levels of anger/aggression (all measured by age 13) were more likely to be arrested for homicide/attempted homicide by age 18. These findings add to the scant scientific literature on the predictors of homicide, and illustrate potential avenues for intervention.

## 1. Introduction

Homicide offending by juveniles is relatively rare, yet approximately 7%–8% of all murders between 2010 and 2014 in the United States involved a juvenile perpetrator [1]. The “all in” average cost to society of a single murder is estimated to exceed $17 million [2]. With homicide being the third highest leading cause of death among 15–24 year olds in the United States in 2014, with the death rate for homicide 10 times higher for Black males (64.7 per 100,000) than white males (6.4 per 100,000), and the rate for Black males being 4.6 times higher than that of Hispanic males (14.1 per 100,000) [3], insight into the prediction of homicide offending among adolescents has obvious repercussions for reducing racial and ethnic disparities. The vast majority of prior work on juvenile homicides has been plagued by methodological limitations, such as small (often convenience and/or clinical) case studies [4,5,6], lack of comparison groups [4,7], or has been comparative/descriptive rather than prospective in nature [8,9]. Clinical case studies most likely overstate the role of mental health in juvenile homicide, lack of comparison groups prevents the ability to elucidate causes of homicide or whether risk factors of homicide differ from those of other offenses, and retrospective studies lend themselves to bias wherein the outcome (homicide offending) is known leading to incomplete or inaccurate recall of self-reported or parent-reported risks [8]. Some prior work has focused on subsequent recidivism among juveniles who have already committed homicide offenses (see References [10,11,12]). However, there is a notable dearth of research on the prospective prediction of a juvenile’s initial homicide arrest. The purpose of the current study is to add to the sparse prior prospective examinations of initial homicide offending through analysis of risk factors prior to age 13 predicting homicide offending by age 18 among male and female youth involved in the juvenile justice system. 

Prior descriptive and retrospective work has highlighted that adverse childhood experiences are common among male juvenile homicide offenders. Specifically, physical abuse [9,13,14], sexual abuse [15,16], chaotic/unstable family environments, such as parental substance abuse, mental health problems, and criminal history [5,6,13,17,18,19,20,21] and witnessing violence [5,14,22] have figured prominently in prior work. Additionally, juveniles’ own mental health problems (including anger, depression, and anxiety) and suicidal ideations have been deemed prevalent among juvenile homicide offenders [6,13,20,23]. Individual-level risk factors among juvenile homicide offenders have included a history of violent offending prior to the homicide offense [13,20], as well as educational deficits and alcohol abuse (in retrospective comparisons to non-violent delinquents; [17,24]). Examining 1354 serious adolescent offenders from the Pathways to Desistance Study, prior work has demonstrated that only lower IQ and exposure to violence predict having been charged with homicide [25]. Examining retrospective criminal histories of adult male habitual criminals, DeLisi and colleagues demonstrated that juvenile confinement predicted subsequent murder arrests, even upon control of a host of other official criminal offending measures, age of onset, and race/ethnicity [26].

DiCataldo and Everrett [4] compared 33 juvenile homicide offenders with 38 violent non-homicidal juveniles, finding that the latter actually possessed a greater number of prominent risk factors than the homicide offenders. Distinguishing homicide offending youth from violent youth was substance abuse at the time of the offense and greater availability of firearms, while no differences were found across groups in parental criminality. Non-homicidal violent juvenile offenders had significantly more prior violent offenses, more mental health problems, self-reported anger issues, and more prior child welfare placements, and were younger at first arrest than the juvenile homicide offenders. Unfortunately, like much of the prior juvenile homicide research, the cross-sectional nature of the study doesn’t allow for causal analysis of the predictors of homicide offending. 

A recent systematic review of 16 (12 case-controls and four longitudinal cohorts) juvenile homicide studies indicated that such studies were a heterogeneous group, but that 10 risk factors appeared consistently across studies [27]. The international review included only higher quality studies, measuring each potential inclusion across selection, sampling, population, measurement, classification, and attrition biases. The largest proportion of included studies suffered from measurement bias (81%) where the validity, reliability, standardization, or inter-rater reliability of the assessments used were unclear, followed by selection-referral bias (occurring where the control group differs from the homicide offender group in terms of risk factors or psychopathology; present in 75% of included studies), though each bias type was present in at least 25% of the studies. The 10 consistent risk factors identified from the included studies were: Low executive function, poor health/illness, epilepsy, violent family members, criminal family members, prior contact with the court, low academic achievement, gang membership, and weapon possession [27]. Risk factors were deemed consistent based on their presence in at least one cohort study and two or more case-control studies, or two or more cohort studies and at least one case-control study [27].

We were able to locate only two prospective studies predicting first-time homicide offending. The first [9] was prospective in that the authors examined whether indicators measured during adolescence differed between those who later commit homicide from those who did not. The study used chi-square analyses to compare two samples; one sample of nine clinically evaluated adolescents who later committed murder (three of which were previously psychiatrically hospitalized in early adolescence, 6 of which received severe head injuries as children), the other of 24 incarcerated juveniles who were neuropsychiatrically evaluated between the ages of 10 and 16 and did not subsequently commit any violent offense within six years of release. Lewis and colleagues [9] found psychotic symptoms and major neurological impairment distinguished subsequent murderers. Physical abuse and serious violence committed as a juvenile were notably not statistically significant. It should be noted that only two of the nine homicide offenders committed the homicide when the subject was still a juvenile (prior to the age of 18).

In a recent prospective analysis of homicide offending, Farrington and colleagues examined predictors (measured between the ages of 7 and 13) of homicide offending among at-risk Pittsburgh Youth Study (PYS) public school males [8,28]. Among the 1512 boys, 37 subsequently committed homicide between the ages of 15 and 29 (2.4% prevalence), with a mean age of conviction of 19.7. Predictive analyses indicated the strongest behavioral risk factors of future homicide offending were suspensions from school and antisocial attitude. While a weak predictor of a future homicide conviction, school truancy was the strongest predictor of a later homicide arrest besides race (with Black males being far more likely [28]). The strongest explanatory risk factors (defined as those indicators that do not measure antisocial behaviors, but are related to them) of homicide offending were living in a disadvantaged neighborhood in childhood and having a young mother (teenager at child’s birth). With relation to criminal history indicators, violent official offending predicted a future homicide arrest, but so did property offending, indicating future homicide offenders were already versatile offenders at a young age. In this first official prospective analysis of juvenile homicide offending among a general population sample (non-clinical), neither early substance use nor peer delinquency independently predicted homicide offending by age 29. Demonstrating the significance of early-onset offending, 70% of the future homicide offenders were first arrested by age 14 compared to 28% of the control group youth (odds ratio = 6.2). An earlier study, using the same PYS males, first predicted violent offenders, and then predicted future homicide among the violent youth [29]. Among the violent offenders, those who commit homicide were more likely to carry a weapon, have a conduct disorder diagnosis, sell hard drugs, have gang-involved fighting, report delinquent peer associations, be held back in school, come from a family on welfare, and be Black youth. 

While the PYS prospective homicide analyses were groundbreaking, lending causal analyses to the study of male homicide offending based on risk factors measured in childhood, the average age of homicide conviction among the future homicide offenders was 19.7, with most homicides committed well past the juvenile years. Additionally, the analyses predicted homicide among at-risk males, though did not distinguish homicide offending among an exclusive justice-involved population of male and female early-onset offenders. As such, the current study aims specifically to prospectively assess the predictors of juvenile homicide offending (by age 18) among a sample of early-onset male and female delinquent youth.

A plethora of criminological research has demonstrated the age of criminal onset is highly predictive of subsequent criminal behavior, and higher levels of frequency and seriousness of offending [30,31,32,33]. Early-onset is typically defined as 12 years old or younger at the time of first offense [34]. These youth are at 2–3 times higher risk for later violence, and are more likely to carry weapons, become affiliated with gangs, and engage in substance use [35,36,37,38]. Early-onset youth have also been demonstrated to have a larger proportion of serious, violent, and chronic (SVC) offending careers [34,35,39] that more likely to persist into adulthood [40]. While these negative repercussions of early-onset offending are well-demonstrated, no prior work has examined the risk factors that predict future homicide offending among early-onset offenders. The current study aims to address that void. 

### The Current Study

The objective of the current study was to examine the constructs that prospectively predict future homicide/attempted homicide offending by the age of 18 among juveniles first arrested at age 12 or younger. Explicitly, we examine the relevance of demographic, criminal history, school, personal and familial, and psychological risk factors in the prediction of homicide/attempted homicide juvenile offending among early-onset juvenile offenders. First, we discuss the data, measures, and analytic strategy, followed by a presentation of the results and our discussion and conclusions.

## 2. Materials and Methods

### 2.1. Participants and Data

The current study uses Florida Department of Juvenile Justice (FDJJ) data on all youth first arrested from 1 December 2006 to 1 December 2008, who were 12 years of age or under at time of that first arrest. The youth must have turned 18 years of age prior to 1 December 2014 to be included. Essentially, this strategy captures the full population of juveniles arrested in Florida in the two years between 1 December 2006 to 1 December 2008, allowing up to 1 December 2014 for the youth to “age out” of the juvenile justice system with respect to new arrests (18 years of age is the age of majority in Florida). There were 9073 youth arrested during the two year study window that fit the requirements of being 12 years of age or younger at first ever arrest, and who turned 18 by 1 December 2014.

Of the 9073 12-year-old, and under, first-time offenders, one youth’s first ever arrest was for a homicide, and no youth were first arrested for attempted homicide. As the purpose of the current study is the prediction of homicide offending, the one youth was removed from the study sample. Additionally, the sample was limited to youth who were assessed by the FDJJ risk/need assessment, the Community Positive Achievement Change Tool (C-PACT) within 90 days of their first ever arrest at age 12 or under. This ensures appropriate time order of using the risk/need assessment information (explained below) in the prediction of subsequent homicide/attempted homicide. Excluding youth without a risk/need assessment within 90 days reduced the sample to 5908 youth. Risk/need assessment information is critical to examining predictors of homicide/attempted homicide offending. Importantly, youth not assessed were those individuals who were never formally processed at a juvenile assessment center. These youths were given a notice to appear in court and the judge and/or prosecuting attorney decided against formal processing (expectedly common as the youth were 12 years of age and under). As our focus is juveniles formally involved in the juvenile justice system, including non-assessed youth would have distorted the pattern of relationships addressed in the current study. The sampling method ensures results presented by the analysis accurately represent prospective prediction of homicide/attempted homicide juvenile offending among early-onset system-involved juvenile offenders (see Appendix A).

Additional data used included all subsequent charge information for each youth prior to the youths’ 18th birthday. The final sample included 5908 youth first arrested prior to age 12, who turned 18 years of age prior to 1 December 2014. The sample was 45.3% Black, and 70.2% male; 24 of the youth were charged with committing homicide/attempted homicide (0.5%) by age 18. The subsequent to first arrest homicides/attempted homicides occurred between the ages of 12.83 (but not the first offense) and 17.95, with the average age being 15.9 when the homicide/attempted homicide was committed.

Data sources were garnered from the FDJJ centralized information system, and include all demographic, referral/charge, and risk/need assessment information. The risk/need assessment, the C-PACT, has been implemented statewide since mid-2006. The C-PACT is validated risk/need assessment administered to all youth formally processed into FDJJ. The assessment is administered by juvenile probation officers who have successfully completed a 3-day training on the assessment, case planning, and the software interface, as well as a standardized 2-day motivational interviewing training. The responses to each item, with the exception of the criminal history items, are based on youth self-report, corroborated with parents/guardians, as well as education and child welfare records (to which the staff have access). The criminal history items are automated into the assessment from the FDJJ centralized information system and are, therefore, not dependent on youth self-report/recall. The C-PACT produces a software-scored risk to re-offend classification (low, moderate, moderate-high, or high) through the forced-choice responses to assessment items. The C-PACT has evidenced predictive validity on par with the established average of such tools in meta-analyses of juvenile offending risk assessment (AUC = 0.64; [41,42]). The inter-rater reliability of the C-PACT, as implemented by the FDJJ, has demonstrated an intra-class correlation coefficient (ICC) of 0.83, with only 4% of items with less than a 75% agreement with an “expert rater” [43]. 

The data that support the findings of this study are available from the Florida Department of Juvenile Justice (data source), but restrictions apply to the availability of these data, which were used under license for the current study, and thus are not publicly available. Data are, however, available from the corresponding author upon reasonable request and with permission of the Florida Department of Juvenile Justice (data source).

### 2.2. Measures

#### 2.2.1. Homicide/Attempted Homicide

The dependent measure assessed whether the youth was charged with homicide or attempted homicide prior to the age of 18 (=1, else = 0). As noted, 24 of the 5908 youth were charged with either homicide (13 youth) or attempted homicide (11 youth) prior to age 18. The most common homicide charges among the 13 youths charged with homicide were “homicide: Murder 1st degree, premeditated” (4 charges) and “homicide: Murder while engaged in certain felony offense” (4 charges), while the 11 youths arrested for attempted homicide were all charged with “homicide: willful kill, attempted felony murder”. Prior work has included both homicide and attempted homicide, as often the only difference between completed and non-completed homicide is the lethality of a gunshot, or the proximity of emergency medical care [2,3,16]. As prospective prediction of juvenile homicide is extremely rare, we include both completed and attempted homicides to increase sample sizes.

#### 2.2.2. Demographic Measures

Demographic measures included sex (male = 1), race/ethnicity, and age at first arrest. Specifically, race/ethnicity dichotomized Black (non-Hispanic) youth from all other race/ethnicities (Black = 1). Hispanic was not included as a separate ethnicity indicator, as it perfectly predicted not committing a future homicide. Age at first arrest was a continuous measure, with all sample youth under the age of 13 at first arrest (range = 10.08–12.99; mean = 12.35).

#### 2.2.3. Presenting Offense Seriousness

The seriousness of the most severe charge of each youth’s initial arrest (at age 12 or younger) was captured according to two dichotomous indicators of whether that first arrest included a violent felony adjudication (yes = 1), or sexual felony adjudication (yes = 1). Violent felonies include referrals involving force or physical harm to another person (against-person offenses), as defined by the Florida Department of Law Enforcement (FDLE) as violent felonies. Felony sexual offenses involve sexual motivation including carnal knowledge, child molestation, communication with a minor for immoral purposes, incest, indecent exposure, indecent liberties, promoting pornography, rape, sexual misconduct, and voyeurism. As could be expected, based on all youths being 12 years of age or younger at first arrest, only 3.9% (231 youths) of the youth’s first arrest included a felony sexual offense.

#### 2.2.4. School Measures

Measures related to the school domain include indicators of conduct, performance, and attendance at the time of first arrest. School measures (as captured in the C-PACT assessment) were based on youth self-report, corroborated with parents/guardians, and educational records (to which the C-PACT assessors have access). Specifically, school conduct was categorized into recognition for good behavior, no conduct problems, problems reported by teachers, calls to parents or police regarding conduct, or whether the youth had been expelled (coded 1–5), with higher values indicative of more conduct problems in the most recent school term (most recent to the first arrest). School performance captured the youth’s academic grades during the most recent school term to the first arrest and was categorized according to grade point average (GPA). Categories included honor students (mostly As), GPA above 3.0, GPA 2.0 to 2.99, GPA 1.0 to 1.99, and GPA below 1.0 (coded 1–5), where higher values indicate worse school performance. Finally, the third school domain indicator assessed school attendance during the most recent term to the first arrest. Attendance was categorized as good attendance/no unexcused absences, some unexcused absences, and habitually truant (coded 0–2), where higher values indicate more attendance problems. Habitually truant is defined as 15 unexcused absences in a 90-day period.

#### 2.2.5. Personal and Familial Risk Factors

Eight personal and familial risk factors were included (as captured in the C-PACT assessment), all assessed at the time of the youth’s first arrest. Antisocial peer association was assessed using a self-report measure of the youth’s current friendship network at the time of first arrest (=1 if youth reported any association with antisocial peers or gang members, else = 0). A dichotomous indicator of whether the youth had a history of court-ordered or voluntary child welfare out-of-home or shelter care placement exceeding 30 days prior to his/her first arrest was included (yes = 1). This indicator was based on self-report, corroborated with child welfare records. Whether the youth was currently using drugs or alcohol within the past six months prior to the first arrest (at age 12 or younger) was captured as a dichotomous indicator (current use = 1). This indicator was based on self-report corroborated with parents/guardians. History of witnessing violence was categorized as to whether the youth had witnessed violence (as witnesses, perpetrators or victims; =1, else = 0) prior to the first arrest. Family violence measured the level of conflict between parents, youth and parents, and among siblings during the six months prior to the first arrest. Family violence was categorized into whether the youth witnessed violence among family members (=1). Instances where the problem history of parents in the household in the six months prior to the first arrest included alcohol or drug problems where classified as having household substance use (=1). This indicator was self-reported by the youth, corroborated by parents/guardians. Problem history of parents in the household which included mental health problems were classified as having household mental illness (=1). Finally, youths whose parents/guardians had a jail or imprisonment history by the time of the youth’s first arrest were classified as having household member incarceration (=1). 

#### 2.2.6. Psychological Risk Factors

Three psychological risk factors were assessed at time of first arrest (as captured in the C-PACT assessment): history of engaging in self-mutilation (=1), formal mental health diagnosis (=1; includes schizophrenia, bi-polar, mood, thought, personality, and adjustment disorders; excludes conduct and oppositional defiant disorders, substance abuse, and attention deficit disorders), history of feelings of anxiety or depression (=1). Additionally, an index of anger issues, and an index of the youth’s level of taking responsibility for his/her actions were included as mental health risk factors. The anger index (α = 0.760) combined indicators of feelings of anger (none, occasional, consistent, or aggressive reactions to anger), verbal aggression (belief such aggression is rarely, sometimes, or often appropriate), physical aggression (belief such aggression is never, rarely, or sometimes/often appropriate). The responsibility index (α = 0.703) combined indicators of the youth’s belief that rules/laws apply to him/her (yes, some rules, rules do not apply, or resents/defiant to rules), and whether the youth accepts responsibility for his/her antisocial behavior (accepts responsibility, minimizes antisocial behavior, or accepts antisocial behavior as appropriate/is proud of antisocial behavior). 

### 2.3. Analysis

The current analysis examines the predictors of homicide/attempted homicide by 18 years of age among a sample of delinquent youth arrested for the first time by age 12. We assess this relationship using a rare events logistic regression model, which is more appropriate than traditional logistic regression when the outcomes of interest, such as those in the current study, are very rare events (<5%). Specifically, given the large number of zeros (non-homicide/attempted homicide charges by age 18) the results of traditional logistic regression may be biased and the predicted probabilities underestimated [44]. For this reason, we utilize a modeling strategy developed by King and colleagues, which takes these issues surrounding rare event data into account. These models have been used in past research on violent offending and homicide victimization [45]. All models presented in the tables below employ the subroutine relogit written for use in STATA [46]. For a detailed discussion of rare events logistic regression, including proofs, we refer readers to King and Zeng [44]. Of note, no correlation between covariates included in regression models exceeded 0.475, suggesting that collinearity was not an issue. 

## 3. Results

Table 1 presents the frequency and percentage of the sample for each categorical measure, and the mean and standard deviations for all continuous measures. As shown, the sample was 45% Black, and 70% male, with 16% arrested for a violent offense at first arrest (by age of 12), and 4% first arrested for a sexual felony. Less than 5% of the sample were honors students at the time of first arrest, with 6% having a GPA below 1.0, and 3% habitually truant. At least some level of antisocial peer association was the norm (63%), with 37% having a history of witnessing violence, 34% living in a household with a parent/guardian who has been to jail/prison, and 12% having a formal mental health diagnosis. These characteristics may be expected as the sample is composed of early-onset youth first arrested under the age of 12. 

Table 2 presents the rare events logistic regression results (odds ratios and 95% confidence intervals). Three models are presented. First, only demographic information and the seriousness of the first arrest (at age 12 or younger) were included (Model 1). As shown, Black youth, and those whose first arrest included a violent (against-person) offense were more likely to be charged with homicide/attempted homicide by age 18. Model 2 adds the school and the personal and familial risk factors as predictors. Household mental illness was significant, with those living in a household with a parent/guardian who has a mental illness 7.4 times more likely to be arrested for homicide/attempted homicide by age 18. Black youth, and those whose first arrest included a violent felony were also more likely, though gender was insignificant in this model. Model 3 includes all predictors, including the psychological risk factors. In this comprehensive model the seriousness of the initial arrest (violent felony) was no longer significant, but the effects for race remained. In addition, Males were 4.7 times more likely, and Black youth 4.2 times more likely to be arrested for homicide/attempted homicide by age 18.

Additionally, as shown in Model 3, household mental illness remained significant with youth residing in a household with a parent/guardian with a mental illness 10.5 times more likely to be arrested for homicide by age 18. The psychological constructs of engaging in self-mutilation and the anger/aggression index also increased the odds of a future homicide/attempted homicide arrest (18.5 and 2.1 times, respectively). None of the school measures (conduct, performance, or attendance) or personal risk factors (peers, child welfare placement, substance abuse, witnessing violence) mattered in the prediction of future homicide in any of our models. Interestingly, the youth’s mental health status (formal diagnosis) at age 12 was not predictive of future homicide/attempted homicide either, nor was whether the youth’s first offense was for a sexual felony. The age at which the youth was first arrested (10 years of age up to before his/her 13th birthday) also did not distinguish future homicide/attempted homicide offenders.

## 4. Discussion

Identification of individual characteristics present among juveniles who go on to commit homicide/attempted homicide in the future is of obvious importance to prevention and intervention scholars as well as for the safety of the general public. Unfortunately, studies examining the prospective prediction of first-time homicide are virtually non-existent (however, see References [8,29]). To our knowledge, the current study is the first prospective study using a sample of male and female adjudicated youth as to what distinguishes those that will commit homicide/attempted homicide by age 18. 

With respect to similarities to prior descriptive and retrospective work, the current study finding of sex (male) being predictive is confirmatory. Discrepant from the Gerard and colleagues [27] systematic review, household violence, parental criminality, the juvenile’s own criminal history, and low academic achievement were not predictive of future homicide in the current study. Perhaps the current sample being all early-onset offending youth, therefore all with some prior criminal history is what differentiates the current results. As prior work has shown the odds of early criminal onset and chronic offending trajectories are both increased by adverse childhood experiences and chaotic families [47], we suspect that sampling methodologies contribute to disparate findings. Additionally, results demonstrate the importance of prospective, longitudinal analyses in the study of juvenile homicide offending.

Our results both confirmed and contradicted the limited prior prospective homicide offending work. Similar to prior work, peer delinquency did not predict future homicide offending, and neither did youth substance use (as measured prior to age 13 in our case). The current finding of males and Black youth at increased odds of future homicide is also in keeping with prior homicide research. Age at first arrest was unrelated to future homicide; we suspect this is due to that fact that our sample was composed completely of youth who were early-onset offenders, having been arrested by age 12. In contrast to the PYS work, school risk indicators (truancy, performance, conduct) were not significant, while such factors figured prominently for the Pittsburgh males. Additionally, finding only household mental illness important in future homicide prediction contradicts prior work highlighting the importance of a host of chaotic family measures. 

Finding household mental illness predictive of homicide offending in the current study and additional chaotic family indicators in prior work points toward the need for prevention targeting the reduction of adverse childhood experience exposures, and targeted, early family therapy and training programs [48], especially within minority and disadvantaged communities [49]. Intervention strategies should include anger management, aggression reduction, and skill-building programs for at-risk youth displaying aggressive behavior [50]. Whether widespread scaling of such prevention and intervention efforts affects juvenile homicide offending rates is an important avenue for future policy and research. Additionally, the relevance of self-mutilation and anger in future juvenile homicide parallels the assumption of the “lockage phenomenon” coined by Mohr and McKnight, which postulates that a proportion of adolescents from very chaotic families tend to react to intense pressure or attempt to escape their environment and familial situation through suicidal or homicidal actions [13]. While descriptive in nature, the Dalby and colleagues study found abused juvenile homicide offenders more likely to have prior suicidal ideations and/or attempts. Future work would benefit from prospectively examining whether extreme (in terms of severity, frequency, and duration) adverse childhood experiences, such as forms of abuse and neglect, moderate the predictors of future homicide.

### Study Limitations

The current study is limited by a lack of socioeconomic and community context measures. Prior work has indicated economic and contextual measures have “washed out” the significance of race in the prediction of homicide and violent offending [51]. It is possible the strong effects of race on homicide offending in the current study would be moderated, or potentially mediated, by inclusion of indicators such as family income/welfare dependence, living in a high-crime neighborhood, and concentrated disadvantage. There is nothing inherent about being Black (or Hispanic, or White, or any other race/ethnicity) that leads a youth to violence, rather it is the presence of risk factors, to which Black youth have been demonstrated to be exposed to at higher prevalence, and to have multiple exposures, than other youth [51,52]. Additionally, these contextual measures have been demonstrated strong predictors of later homicide in the PYS sample, though were stronger for future homicide conviction than arrest [8,28]. 

Additional limitations include the lack of victim information, as well as a need for future research to conduct sex-specific analyses, as prior work has shown girls are more likely to kill family members and perform secondary roles with male accomplices in homicide offending [5]. Furthermore, the current study lacked indicators of weapon carrying, which figured prominently in the PYS prospective homicide prediction studies [8,28,29]. Additionally, unlike the PYS studies, the current study included only juvenile homicide offending. As the PYS studies indicated, many of the future homicides are committed after 18 years of age. However, we argue prediction of future juvenile homicide offending based on early adolescent indicators is relevant in its own right, especially to juvenile justice professionals.

## 5. Conclusions

This study examined the extent to which demographic, criminal history, school, personal and familial, and psychological risk factors measured by age 12 predict homicide offending up to age 18. As stated in prior work, “these questions are important because they identify the early presence of risk factors several years prior to the murder taking place” [8] (p. 57). The current study expanded our knowledge of prospective homicide prediction beyond the PYS analyses of inner-city public school males by examining the predictors (measured prior to age 13) of juvenile homicide offending (homicide offending by age 18) among male and female juvenile justice-involved youth. The current study has attempted to add to the sparse research base regarding prospective prediction of juvenile homicide. It is our hope the field progresses beyond comparative and retrospective analyses toward the understanding of indicators predictive of future extreme violence, with future work demonstrating whether recommended intervention can prevent juvenile homicide among at-risk youth.

## Figures and Tables

**Table 1:** Descriptive statistics for the analysis of prospective homicide offenders (*n* = 5908).

Categorical Measures	Frequency	%
Black	2678	45.3
Male	4149	70.2
*Presenting Offense Seriousness*		
Violent Felony	952	16.1
Sexual Felony	231	3.9
*School Measures*		
School conduct		
Recognition for good behavior	410	6.9
No problems with school conduct	2469	41.8
Problems reported by teachers	1141	19.3
Calls to parents of police	1823	30.9
Dropped out/expelled	65	1.1
Academic performance		
Honor Student	252	4.3
GPA above 3.0	1075	18.2
GPA 2.0-3.0	2788	47.2
GPA 1.0 to 2.0	1454	24.6
GPA below 1.0	339	5.7
School attendance		
Good attendance	4269	72.3
Some unexcused absences	1468	24.8
Habitually truant	171	2.9
*Personal and Familial Risk Factors*		
Antisocial peer association	3694	62.5
History of child welfare placement	464	7.9
Substance abuse history	302	5.1
History of witnessing violence	2183	37.0
Family violence history	690	11.7
Household substance abuse history	339	5.7
Household mental illness history	86	1.5
Household member incarceration history	2034	34.4
*Psychological Risk Factors*		
Self-mutilation history	45	0.76
Mental health problem history	694	11.7
Anxiety/depression history	1347	22.8
**Continuous Measures**	**Mean**	**Standard Deviation**
Age at first referral	12.34	0.558
Anger Index	0.0	0.822
Responsibility Index	0.0	0.878

**Table 2:** Rare-event logistic regression results for the analysis of prospective homicide offenders (*n* = 5908).

Variables	Model 1		Model 2		Model 3	
	OR	95% CI	OR	95% CI	OR	95% CI
Gender	4.093	[0.951–17.612]	3.756	[0.789–17.868]	4.747 *	[1.075–20.959]
Black	4.187 **	[1.548–11.323]	4.127 **	[1.595–10.676]	4.178 **	[1.611–10.832]
Age at first referral	1.372	[0.626–3.006]	1.247	[0.542–2.869]	1.289	[0.538–3.089]
Violent felony	2.663 *	[1.092–6.497]	2.606 *	[1.032–6.577]	2.354	[0.943–5.879]
Sex felony	0.624	[0.075–5.184]	0.726	[0.093–5.679]	0.790	[0.103–6.063]
School conduct			0.721	[0.420–1.239]	0.611	[0.343–1.086]
Academic performance			1.399	[0.839–2.334]	1.318	[0.784–2.215]
School attendance			1.684	[0.874–3.242]	1.422	[0.694–2.910]
Antisocial peers			2.218	[0.786–6.262]	2.111	[0.733–6.079]
Child welfare placement			0.718	[0.090–5.718]	0.720	[0.086–6.057]
Substance abuse			1.159	[0.146–9.183]	0.948	[0.126–7.136]
Witnessed violence			1.249	[0.517–3.014]	1.078	[0.427–2.722]
Family violence			1.266	[0.349–4.598]	1.036	[0.279–3.843]
Household substance abuse			1.379	[0.270–7.044]	1.468	[0.254–8.473]
Household mental illness			7.356 *	[1.397–38.726]	10.504 *	[1.582–69.745]
Household incarceration			1.443	[0.611–3.412]	1.292	[0.507–3.288]
Self-mutilation					18.468 *	[1.654–206.229]
Mental health problems					0.379	[0.032–4.431]
Anger index					2.084 **	[1.213–3.580]
Anxiety					0.750	[0.268–2.101]
Responsibility index					1.024	[0.672–1.558]
Constant	0.000 *	[0.000–0.248]	0.000 *	[0.000–0.430]	0.000	[0.000–1.209]
R-Squared (Nagelkerke)	0.077		0.119		0.160	

OR = Odds Ratio; CI = 95% Confidence Interval; * *p* < 0.10, ** *p* < 0.05.

## Appendix A

The youth with risk/need assessments within 90 days of first arrest were less Black (45.3%, compared to 49% for those without a C-PACT assessment), and more male (70.2%, compared to 65.9%) than excluded youth. Both groups were composed of 14% Hispanic, and the average age at first arrest for both groups was 12.3 years of age. With respect to presenting offense of the first ever arrest, the most prominent charges for both groups (included and excluded youth) were assault/battery (21% for included youth, compared to 30% for youth without a C-PACT assessment) and petit larceny (18%, compared to 26%). However, included youth had burglary (13%) and aggravated assault/battery (10%) as their 3rd and 4th most prominent charges, whereas youth without a C-PACT had disorderly conduct (11%) and vandalism (6%), indicating the included youth were potentially a slightly more serious sample.
